# Global research hotspots and trends in the application of artificial intelligence in gastric cancer: a bibliometric analysis from 2005 to 2024

**DOI:** 10.3389/fonc.2025.1591655

**Published:** 2025-10-17

**Authors:** Lu Chen, Jinying Zhao, Zijian Liu, Lingzu Kong, Dan Zhou, Fuchun Wang

**Affiliations:** ^1^ College of Acupuncture and Tuina, Changchun University of Chinese Medicine, Changchun, Jilin, China; ^2^ Acupuncture Clinical Center, Affiliated Hospital of Changchun University of Chinese Medicine, Changchun, Jilin, China

**Keywords:** artificial intelligence, gastric cancer, bibliometrics, CiteSpace, VOSviewer

## Abstract

**Background:**

Gastric cancer (GC) is a prevalent gastrointestinal malignancy. In recent years, the application of artificial intelligence (AI) in GC has become increasingly widespread. This study aims to employ bibliometric analysis to offer valuable insights for researchers.

**Methods:**

Publications concerning the application of AI in GC between 2005 and 2024 were retrieved from the Web of Science Core Collection. Subsequently, VOSviewer, CiteSpace, and Scimago Graphica were employed to conduct the bibliometric analysis of the selected literature.

**Results:**

A total of 903 publications were included in this study. In the past two decades, the application of AI in GC has become more widely used, and the number of papers published has shown a rapid growth trend. China, Japan, and South Korea are the most prolific countries in this field. Yonsei University, the Chinese Academy of Sciences, and Shanghai Jiao Tong University are the three institutions with the most publications. *Surgical Endoscopy and Other Interventional Techniques* is the most published journal and also the most cited journal. Woo Jin Hyung from Yonsei University is both the most prolific author and the author with the highest H-index. Gastric cancer, surgery, and artificial intelligence are the three keywords most used. The keywords “upper gastrointestinal endoscopy” and “artificial intelligence” have been prominent until now.

**Conclusion:**

This study offers a comprehensive visual overview of the application of AI in GC over the past two decades. AI-assisted screening, diagnosis, and prognosis prediction in GC are anticipated to represent focal points of future research in this domain.

## 1 Introduction

Gastric cancer (GC) is currently recognized as the fifth most frequently diagnosed form of malignancy worldwide and stands as the fourth primary contributor to deaths associated with cancer. In 2020, approximately 1.09 million new cases and 770,000 deaths were reported (1). By 2040, the incidence of GC is projected to reach 1.8 million new cases, with mortality rising to 1.3 million worldwide (2). Early detection of GC remains difficult because of the insidious and non-specific nature of early symptoms, which frequently leads to delayed diagnosis (3, 4). The 5-year survival rate for patients with advanced GC is reported to be only 22% (5). Therefore, enhanced screening and management strategies are essential to improve both the quality of life and survival outcomes of patients with GC.

Artificial intelligence (AI) is a technology that simulates human intelligence through computer programs, including deep learning (DL) and machine learning (ML). AI-assisted systems, such as auxiliary examination and diagnostic systems, demonstrate excellent performance in the diagnosis and screening of GC and have garnered significant attention in the diagnosis and treatment of GC (6). AI-assisted systems can promptly detect and identify subtle abnormalities in radiological, pathological, and endoscopic images, differentiate cancerous from non-cancerous lesions, and enhance the ability to detect and diagnose GC (7). Studies have demonstrated that AI diagnostic models exhibit high accuracy in detecting gastrointestinal cancers, with sensitivity comparable to that of expert endoscopists and superior to non-expert endoscopists (8). Furthermore, AI technology has proven effective in diagnosing the depth of invasion of early gastric cancer (EGC) with high accuracy, sensitivity, and specificity (9). This not only improves the detection rate of EGC but also enhances the accuracy of GC diagnosis, facilitates timely treatment and care, and further increases the survival rate of GC patients (10). Additionally, AI has demonstrated effectiveness in the diagnostic staging of gastric cancer (11), survival prediction (12), and risk prediction (13), providing crucial support for prognosis decision-making and care of GC. In conclusion, AI holds significant potential in assisting with GC screening, diagnosis, and prognosis prediction (14, 15). The collaboration between clinicians and AI systems will result in a highly complementary, more efficient, and accurate management of GC, benefiting the majority of GC patients.

Through quantitative evaluation of scholarly outputs within a defined field of study, bibliometric analysis enables the detection of collaborative trends across nations, research organizations, and individual scholars. It enables the extraction of key information from large datasets and offers an effective means for newcomers or interdisciplinary researchers to understand the developmental trajectory and current state of the field (16). In recent years, as research on the application of AI in the diagnosis and treatment of GC has increased, scholars have encountered difficulties in comprehending this field. To the best of our knowledge, a systematic bibliometric analysis of this field is still lacking. Unlike previous reviews that focus on specific technologies or clinical problems, this study employs a systematic bibliometric analysis method to comprehensively examine research on the application of AI in GC over the past 20 years, using a unique and visual network map to identify current research hotspots and emerging trends, thereby providing valuable insights for the future development of this field.

## 2 Materials and methods

### 2.1 Literature search and screening

**Figure 1:**
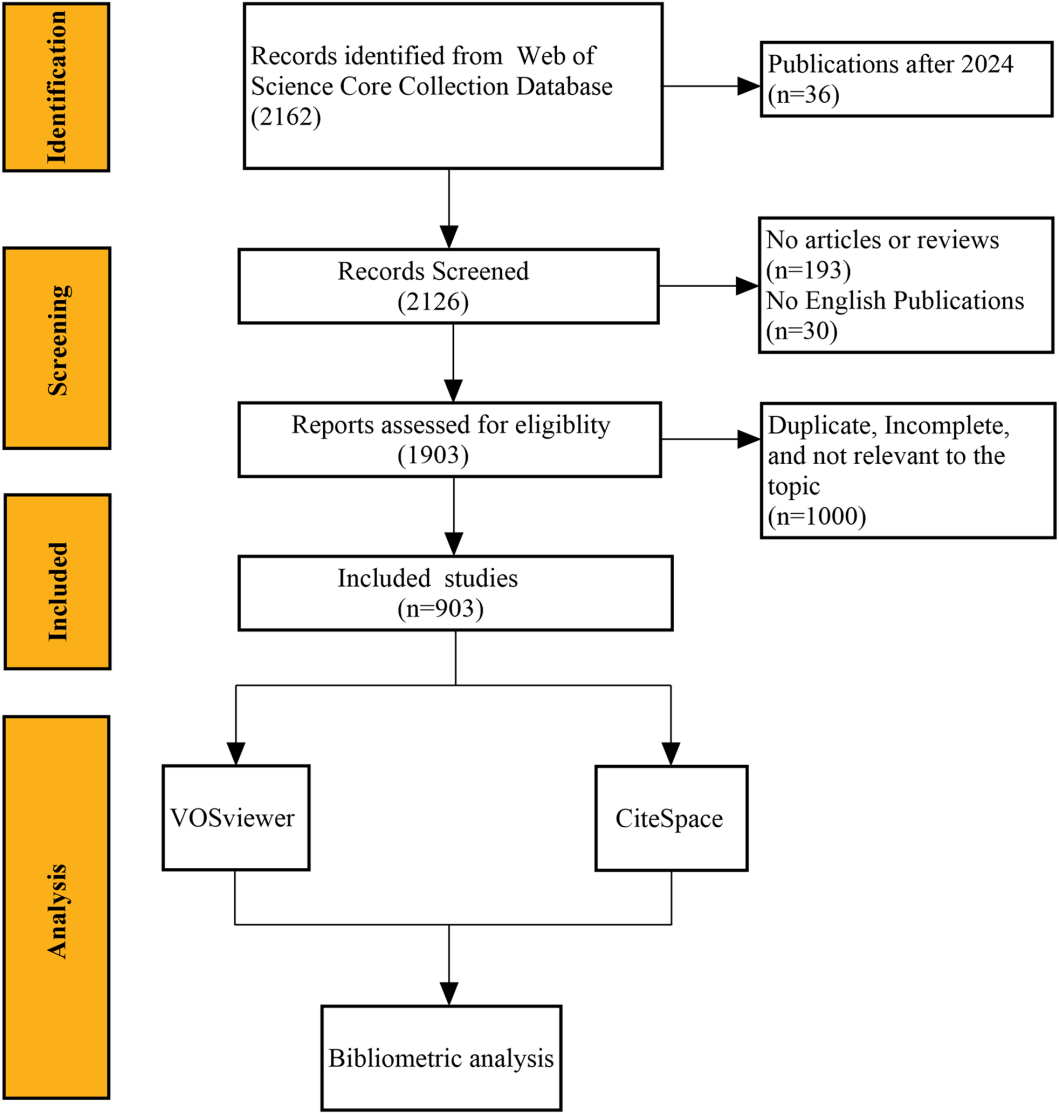
Flow chart of literature screening.

The Web of Science Core Collection (WoSCC) was chosen for this study because it covers more than 12,000 academic journals, enables extensive retrieval of relevant literature, and is widely used by researchers for bibliometric analysis (17–19). Literature about the application of AI in GC from 2005 to 2024 was retrieved from the WoSCC. The search strategy employed the following topic search (TS) terms: (“Stomach Neoplasm” OR “Gastric Neoplasm” OR “Cancer of Stomach” OR “Gastric Cancer” OR “Stomach Cancer”) AND (“Artificial Intelligence” OR “Computational Intelligence” OR “Machine Intelligence” OR “Computer Reasoning” OR “AI” OR “Computer Vision System” OR “Knowledge Acquisition” OR “Knowledge Representation” OR “neural network*” OR “machine learning” OR “deep learning” OR “natural language processing” OR “robot*”). For further analysis of the content, inclusion and exclusion criteria were established. Inclusion criteria were as follows: (1) research consisting of reviews and articles; (2) studies written in English. Exclusion criteria were as follows: (1) studies that did not meet the inclusion criteria; (2) research topics unrelated to AI and GC; (3) studies for which the full text could not be obtained; (4) duplicate publications. Two researchers independently performed the screening, and any disputes were resolved through discussion with a third researcher. Meanwhile, the remaining literature was exported in plain text format for the next analysis. **Figure 1** shows the data collection and screening process.

### 2.2 Data analysis

The main software used in this study includes Microsoft Office Excel 2021, Origin 2024, CiteSpace 6.4 (20), VOSviewer 1.6.20 (21), and Scimago Graphica. Specifically, Microsoft Office Excel was utilized to organize the data, and Origin was employed to map annual publication trends. The software VOSviewer was utilized to generate visualized network maps illustrating the relationships among countries, authors, and academic journals. In these diagrams, node size is directly proportional to the frequency of occurrence; larger nodes indicate higher frequencies, while smaller nodes represent lower frequencies. The connecting lines between nodes reflect the strength of association between them. In addition, total link strength (TLS) serves as a key metric employed to assess the influence of a node within the overall network. The TLS of a node is defined as the sum of the link strengths between the node and all other nodes to which it is directly connected. Scimago Graphica was utilized to map the geographical distribution of countries or regions where articles were published, thereby visualizing cooperative relationships among them. Furthermore, CiteSpace was used to generate the co-occurrence network of institutions, references, and keywords, along with the keyword cluster map and keyword emergence map.

## 3 Results

### 3.1 Trends in annual publication volume

**Figure 2:**
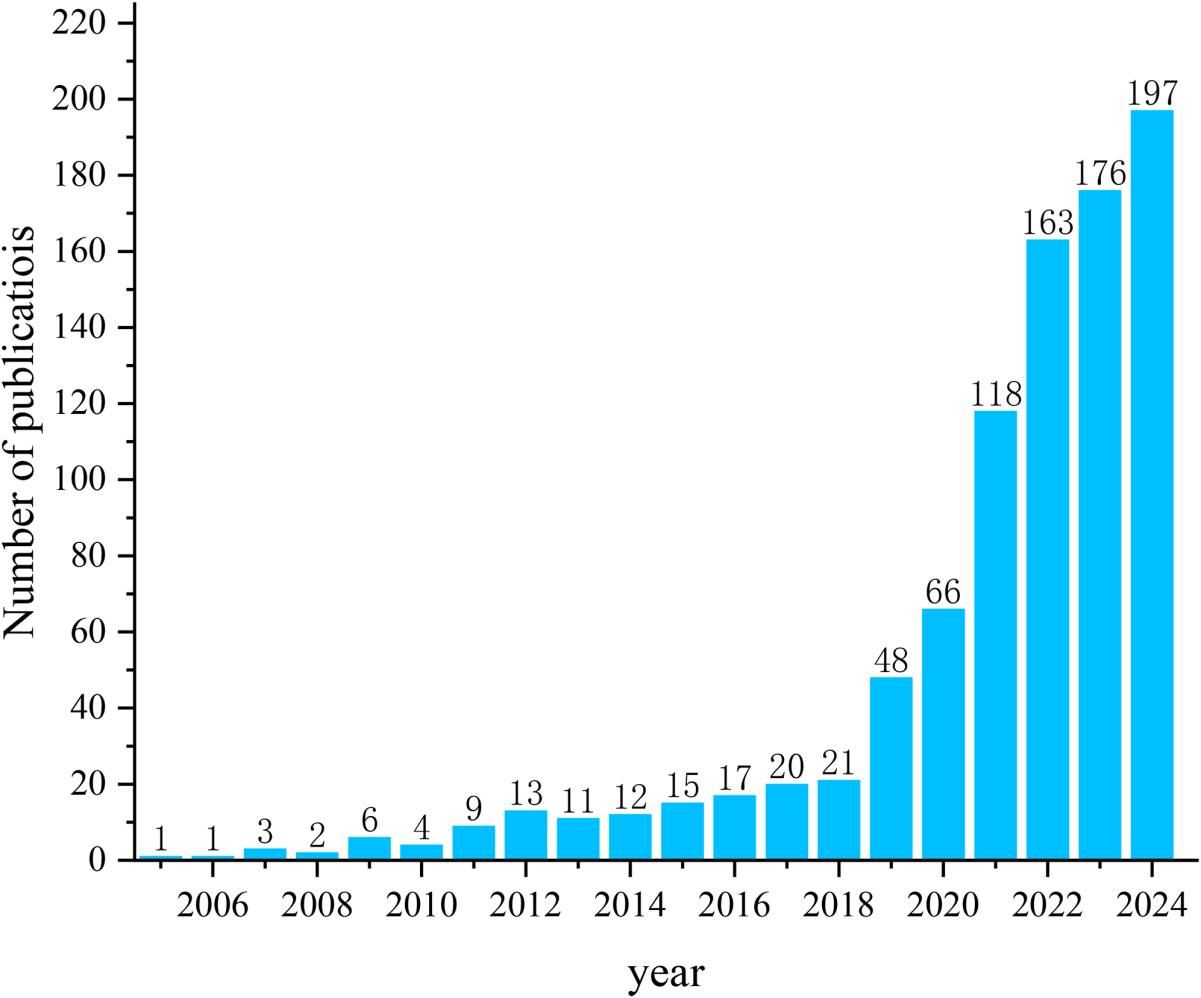
Trends in the number of publications.

A total of 903 articles were included (**Figure 2**). Among these, 764 articles and 139 reviews were included. The number of publications has steadily increased from one publication in 2005 to 21 publications in 2018. After that, the number of publications on AI applications in GC grew rapidly over the next 6 years, reaching 197 in 2024. The number of publications in this field is expected to continue to grow in the future.

### 3.2 Analysis of countries or regions of publications

**Figure 3:**
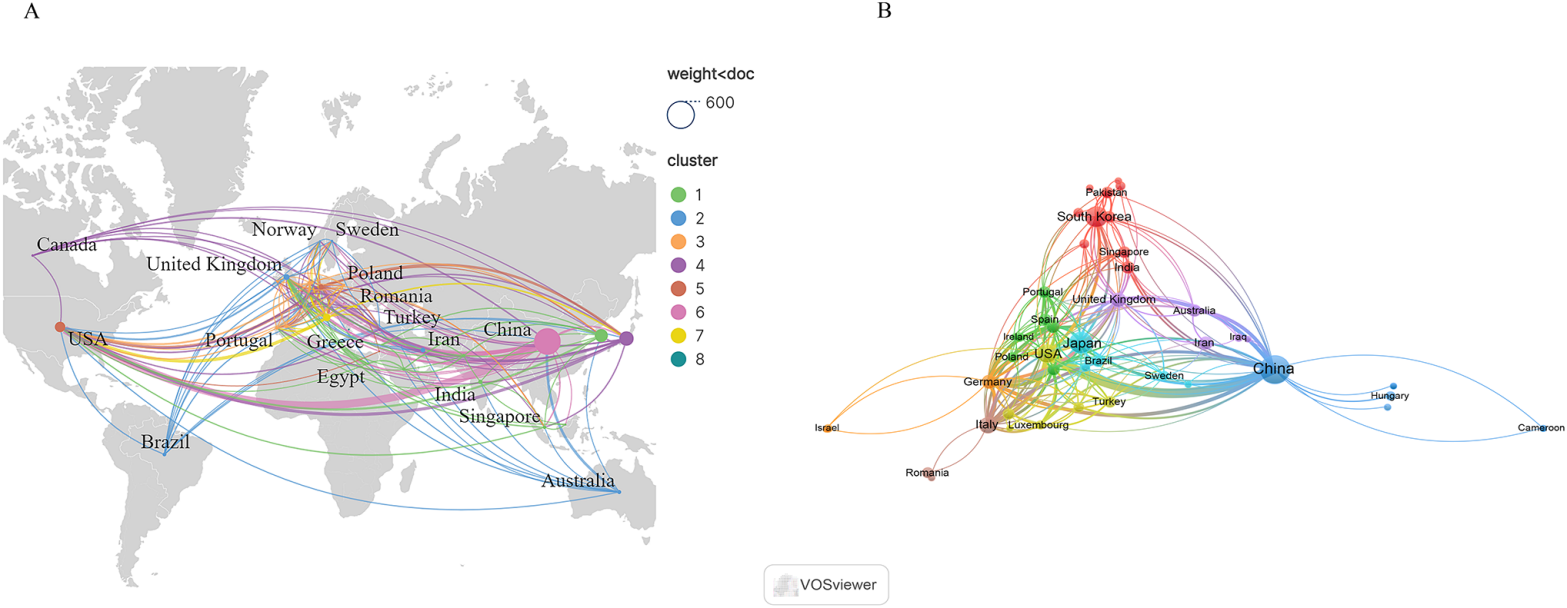
**(A)** Geographic distribution of countries/regions. **(B)** Cooperation networks between countries.

A total of 51 countries or regions were found to have engaged in research within this domain. **Figure 3** presents the geographic distribution of contributing countries or regions, while **Figure 3** depicts the network of international cooperation. As shown in **Table 1**, China was ranked first with 497 publications, followed by Japan (146), Korea (120), and the United States (77). The remaining countries or regions each contributed fewer than 40 publications. Notably, China and the United States exhibited the highest TLS, suggesting stronger collaborative relationships with other countries.

**Table 1:** Top 10 countries/regions of publications.

Rank	Country/region	Frequency	Centrality	TLS
1	China	497	0.51	115
2	Japan	146	0.03	61
3	South Korea	120	0.14	32
4	USA	77	0.18	109
5	Italy	39	0.17	73
6	United Kingdom	25	0.18	65
7	Germany	21	0.19	88
8	Iran	15	0.07	7
9	Netherlands	14	0.02	51
10	India	13	0.01	8

### 3.3 Analysis of institutions

**Figure 4:**
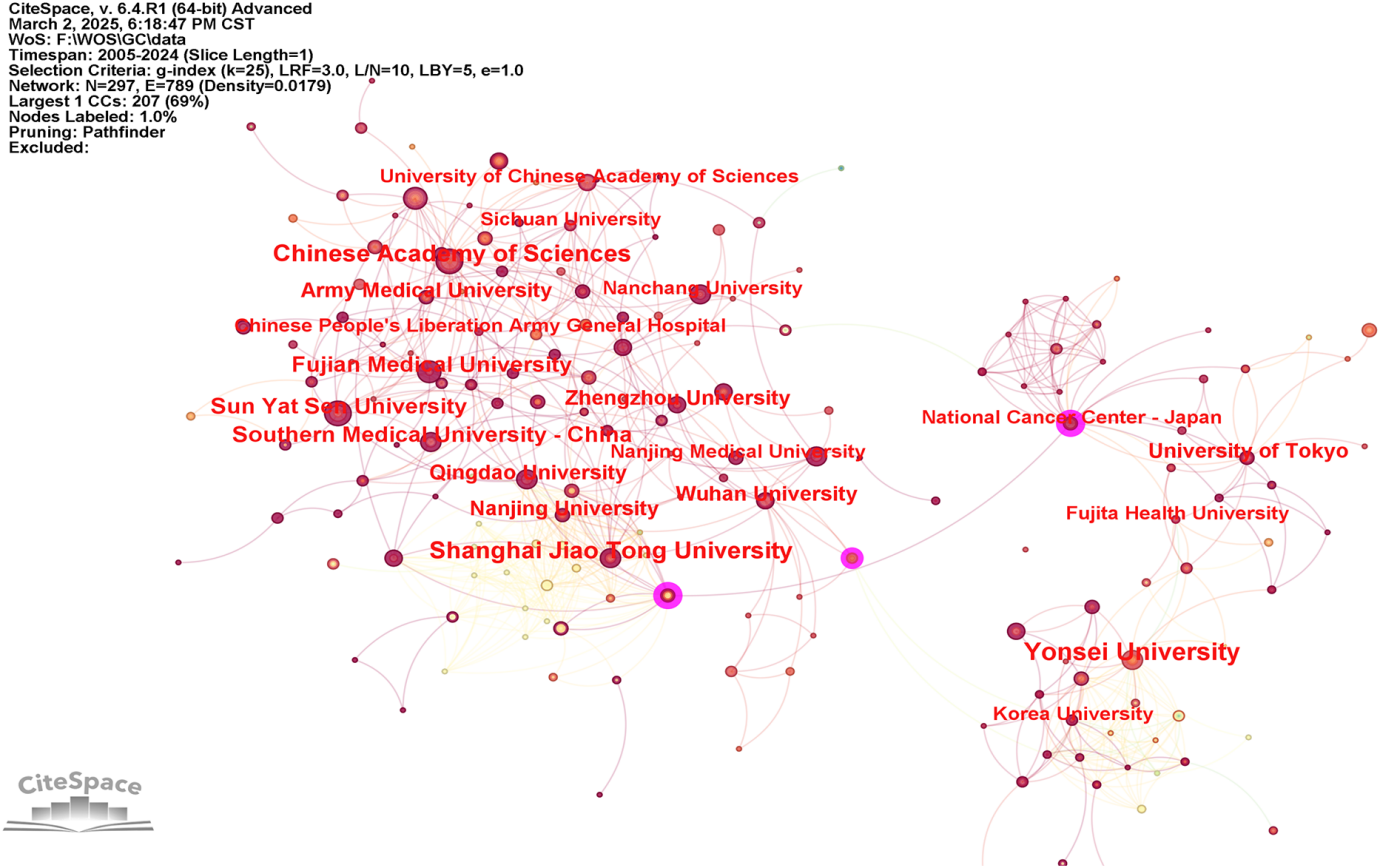
Institutional co-occurrence network map.

To examine institutional contributions to the application of AI in GC, an analysis was conducted of publication counts by institution. In total, 1,278 research institutions across the globe were recognized as active contributors within this domain. The collaborative relationships among these institutions are visualized in **Figure 4**, whereas **Table 2** provides the ranking of the ten institutions with the most substantial publication output. As illustrated, Yonsei University, the Chinese Academy of Sciences, and Shanghai Jiao Tong University accounted for the highest numbers of publications, with 44, 35, and 35, respectively. Furthermore, the National Cancer Center – Japan, Azienda Ospedaliero Universitaria Careggi, Huazhong University of Science and Technology, Tianjin Medical University, and Fudan University were distinguished by centrality values equal to or exceeding 0.1.

**Table 2:** Top 10 institutions in terms of publications.

Rank	Institutions	Country/region	Frequency	Centrality
1	Yonsei University	Korea	44	0.07
2	Chinese Academy of Sciences	China	35	0.09
3	Shanghai Jiao Tong University	China	35	0.05
4	Fujian Medical University	China	29	0.05
5	Sun Yat-sen University	China	27	0.04
6	Southern Medical University	China	27	0
7	University of Tokyo	Japan	23	0.04
8	Wuhan University	China	22	0.08
9	Army Medical University	China	22	0.06
10	Nanjing University	China	22	0

### 3.4 Analysis of journals

**Figure 5:**
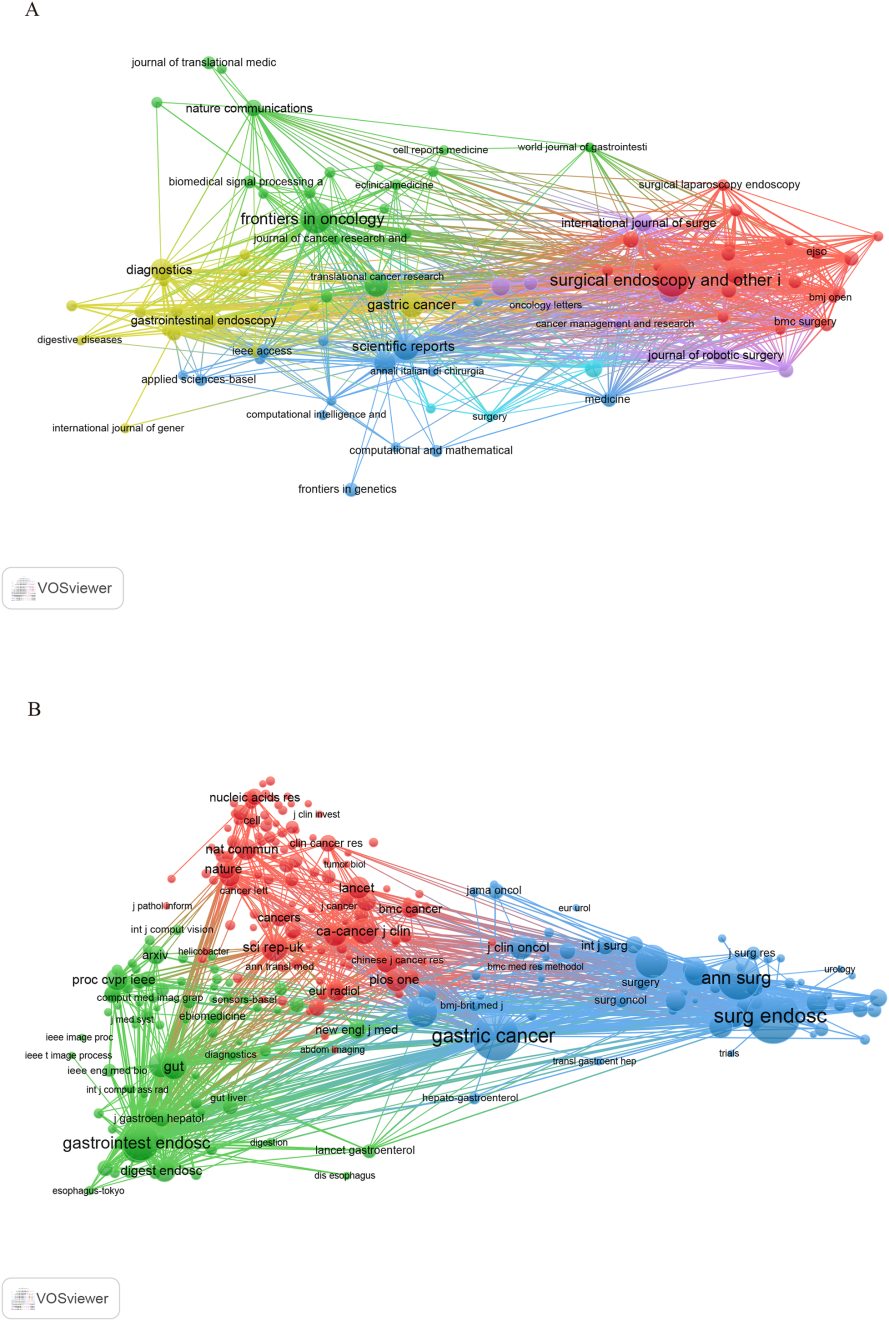
**(A)** Visualization network diagram of journals. **(B)** Network diagram of cited journal visualization regarding the application of AI in GC.

This study includes 903 papers published in 282 journals. **Figure 5** depicts the network visualization of journals involved in the dissemination of research, while **Table 3** lists the ten leading journals ranked by publication count alongside their most recent impact factors. According to the data presented in **Table 3**, the journal *Surgical Endoscopy and Other Interventional Techniques*, published in the United States, contributed the largest volume of articles in this field, amounting to 57 papers. This was followed by *Frontiers in Oncology* (45) and *Scientific Reports* (31). Impact factor and Journal Citation Reports (JCR) quartile rankings are important indicators for assessing the academic influence of scholarly journals. According to the JCR classification published by Clarivate, journals are categorized into four quartiles: Q1, Q2, Q3, and Q4. Among the journals analyzed, only three—*Surgical Endoscopy and Other Interventional Techniques*, *Frontiers in Oncology*, and the *Journal of Gastrointestinal Surgery*—were placed in Q2, while the remaining seven journals were classified as Q1. It is worth noting that all ten journals had impact factor scores below 7.

**Table 3:** Top 10 journals in terms of publications.

Rank	Journals	Country	Counts	Division	IF (2023)
1	Surgical Endoscopy and Other Interventional Techniques	USA	57	Q2	2.4
2	Frontiers in Oncology	Switzerland	45	Q2	3.5
3	Scientific Reports	United Kingdom	31	Q1	3.8
4	Gastric Cancer	Japan	25	Q1	6.0
5	Cancers	Switzerland	24	Q1	4.5
6	Diagnostics	Switzerland	20	Q1	3.0
7	Annals of Surgical Oncology	USA	19	Q1	3.4
8	World Journal of Gastroenterology	USA	19	Q1	4.3
9	Journal of Gastrointestinal Surgery	USA	15	Q2	2.2
10	Gastrointestinal Endoscopy	Switzerland	14	Q1	6.7

**Figure 5** illustrates the visualization network of cited journals with at least twenty citations. As shown in **Table 4**, *Surgical Endoscopy and Other Interventional Techniques* (2170) was identified as the most frequently cited journal. It was followed by *Gastric Cancer* (1696) and *Annals of Surgery* (1300). The cited journals were classified in Q2, except for *Surgical Endoscopy and Other Interventional Techniques*, whereas the remaining nine journals were classified in Q1. Notably, *CA: A Cancer Journal for Clinicians* and *Gut* exhibited high levels of activity in this field, with impact factors of 503.1 and 23, respectively.

**Table 4:** Top 10 cited journals.

Rank	Cited Journals	Country	Counts	Division	IF (2023)
1	Surgical Endoscopy and Other Interventional Techniques	USA	2170	Q2	2.4
2	Gastric Cancer	Japan	1696	Q1	6.0
3	Annals of Surgery	USA	1300	Q1	7.5
4	Gastrointestinal Endoscopy	USA	929	Q1	11.5
5	Annals of Surgical Oncology	USA	744	Q1	3.4
6	Endoscopy	Germany	618	Q1	11.5
7	World Journal of Gastroenterology	USA	617	Q1	4.3
8	Scientific Reports	United Kingdom	450	Q1	3.8
9	Ca-a Cancer Journal For Clinicians	USA	429	Q1	503.1
10	Gut	United Kingdom	408	Q1	23.0

### 3.5 Analysis of authors of publications

A total of 5,095 authors were involved in research related to this field. According to Price’s Law (22), the threshold for identifying core authors is established through the following formula, which specifies the minimum number of required publications:


$$m=0.749\times \sqrt{n_{\max}}$$

**Figure 6:**
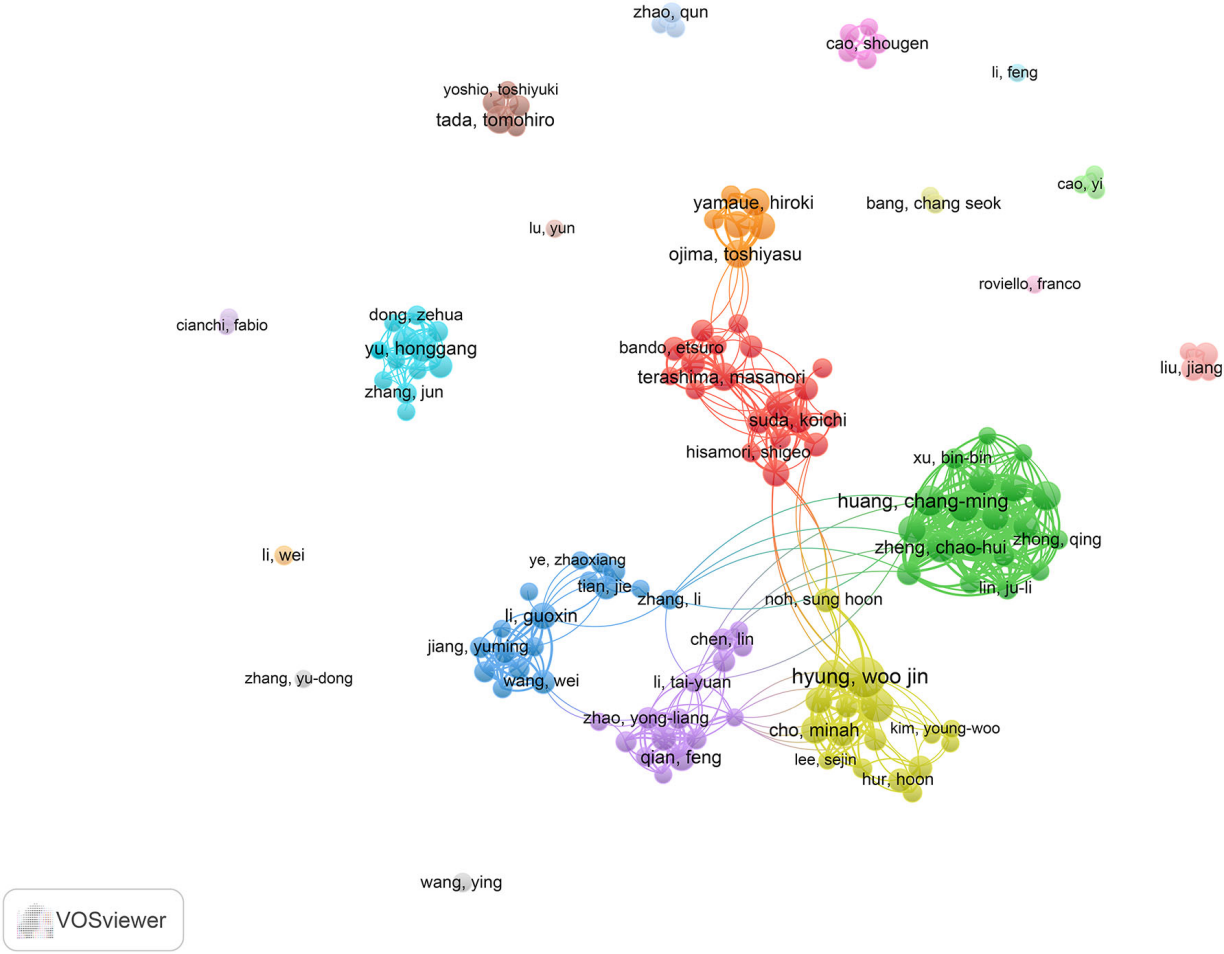
Map of the core authors’ collaborative network.

In this context, n_max_ denotes the publication count of the most prolific contributor. Accordingly, individuals with 5 or more published works were designated as core authors, leading to the identification of 135 such contributors. As shown in **Table 5**, Woo Jin Hyung is the most prolific author with 35 publications and an H-index of 71. Following him are Hyoung-Il Kim (21 publications, H-index = 54) and ChangMing Huang (18 publications, H-index = 39). **Figure 6** presents the co-authorship network of researchers with at least 5 publications, illustrating the patterns of collaboration among leading contributors in the field. The magnitude of each node reflects the volume of publications it represents, whereas the coloration of nodes and their connecting links denotes the clustering to which they belong. Notably, the 135 core authors with 5 or more publications form 7 larger clusters.

**Table 5:** Top 11 authors of publications.

Rank	Author	Documents	Citations	H-index
1	Woo Jin Hyung	35	1445	71
2	Hyoung-Il Kim	21	525	54
3	Chang-Ming Huang	18	275	39
4	Ping Li	17	279	43
5	Chao-Hui Zheng	17	271	39
6	Qi-Yue Chen	16	271	37
7	Minah Cho	15	292	20
8	Toshiyasu Ojima	15	229	26
9	Taeil Son	15	480	31
10	Tomohiro Tada	15	936	31
11	Masanori Terashima	15	457	58

### 3.6 Analysis of cited references

**Figure 7:**
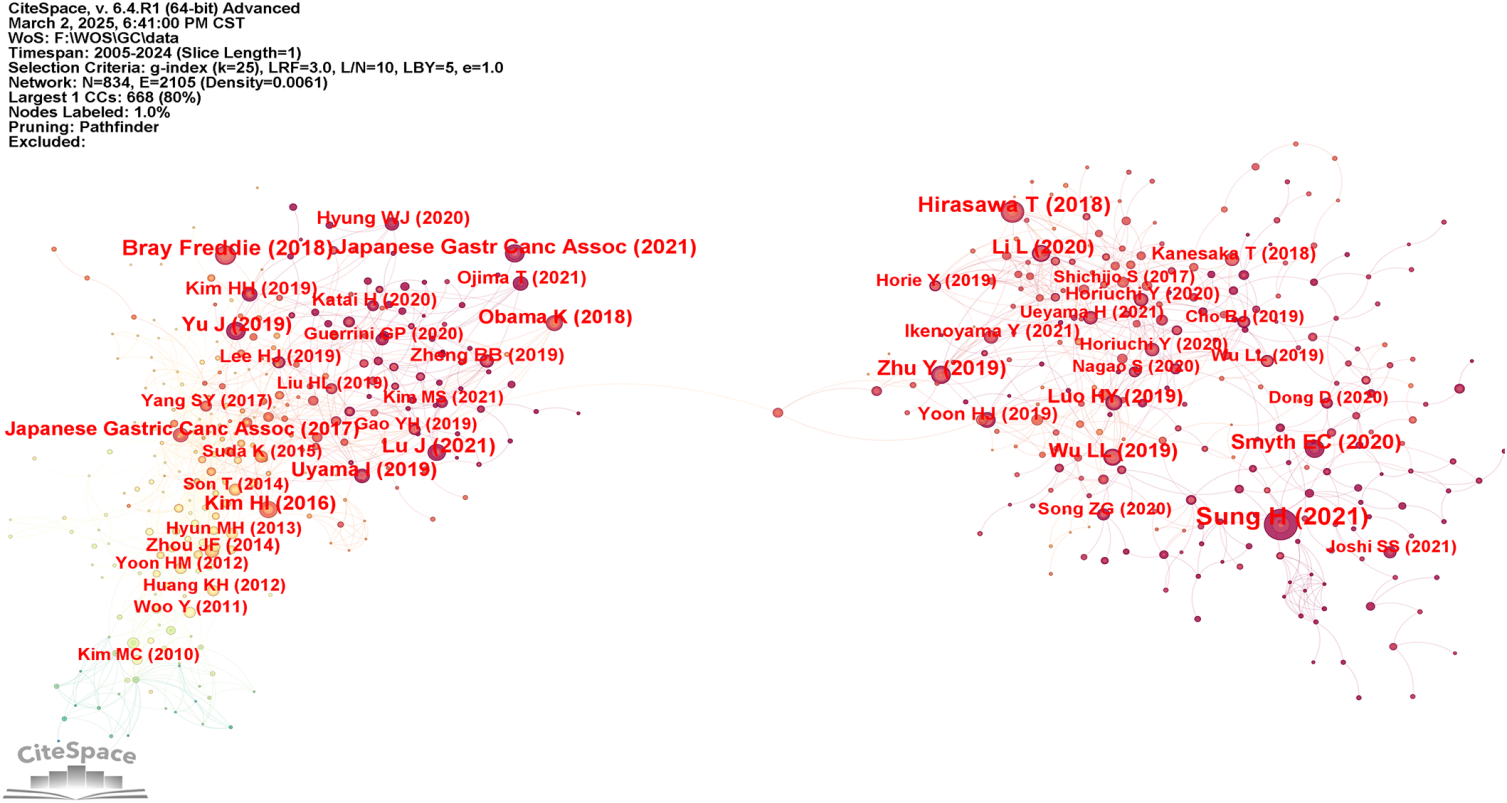
Graph of co-occurrence network of cited references.

The analysis of co-cited references provides an essential basis for advancing disciplinary research, as it enables a more precise delineation of the central themes within the field. Using VOSviewer, a co-citation network of cited references was generated (**Figure 7**), while **Table 6** displays the ten most frequently co-cited works, each referenced on more than sixty occasions. Notably, two articles provided comprehensive assessments of global cancer incidence and mortality in 2018 and 2020, respectively. The findings indicated that cancer continues to pose a major global public health challenge, and effective measures for prevention, detection, and treatment are considered essential for effective cancer control (1, 23). In addition, two other articles demonstrated the accuracy and specificity of AI in diagnosing GC and assessing the depth of tumor invasion (24, 25). In summary, these studies primarily addressed the epidemiology, diagnosis, and prognosis of GC.

**Table 6:** Top 10 cited references.

Rank	Cited reference	First author	Counts	Year
1	Global Cancer Statistics 2020: GLOBOCAN Estimates of Incidence and Mortality Worldwide for 36 Cancers in 185 Countries	Sung H	199	2021
2	Application of artificial intelligence using a convolutional neural network for detecting gastric cancer in endoscopic images	Hirasawa T	99	2018
3	Global cancer statistics 2018: GLOBOCAN estimates of incidence and mortality worldwide for 36 cancers in 185 countries	Bray Freddie	91	2018
4	Application of convolutional neural network in the diagnosis of the invasion depth of gastric cancer based on conventional endoscopy	Zhu Y	80	2019
5	Japanese gastric cancer treatment guidelines 2018 (5th edition)	Japanese Gastric Cancer Association	78	2021
6	Assessment of Robotic Versus Laparoscopic Distal Gastrectomy for Gastric Cancer: A Randomized Controlled Trial	Lu J	68	2021
7	Gastric cancer	Smyth EC	68	2020
8	Clinical advantages of robotic gastrectomy for clinical stage I/II gastric cancer: a multi-institutional prospective single-arm study	Uyama I	67	2019
9	Multicenter Prospective Comparative Study of Robotic Versus Laparoscopic Gastrectomy for Gastric Adenocarcinoma	Kim HI	64	2016
10	Effect of Laparoscopic vs Open Distal Gastrectomy on 3-Year Disease-Free Survival in Patients With Locally Advanced Gastric Cancer: The CLASS-01 Randomized Clinical Trial	Yu J	64	2019

### 3.7 Analysis of keywords

**Figure 8:**
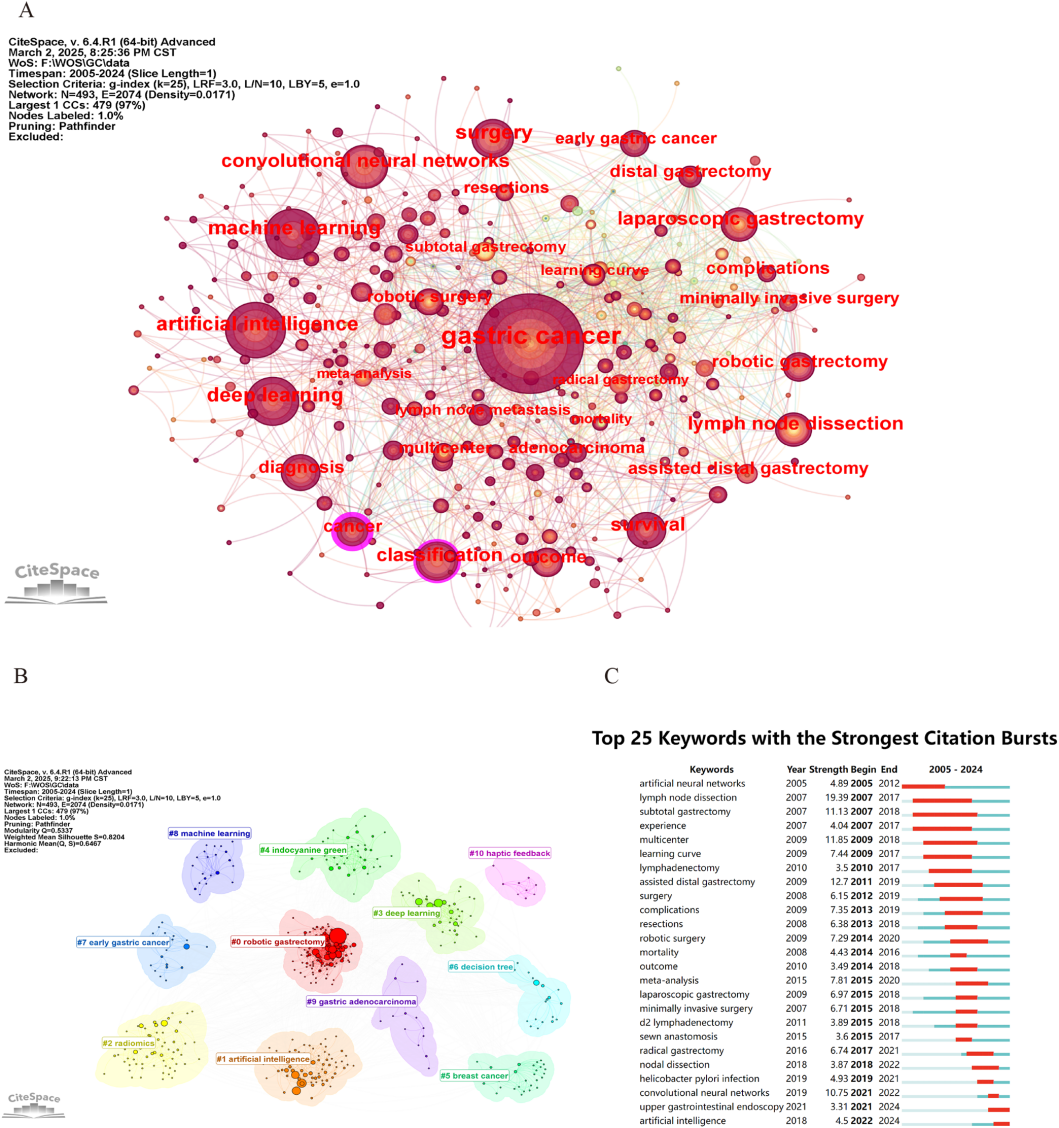
**(A)** Keyword co-occurrence visualization. **(B)** Clustering representation of keywords. **(C)** Top 25 keywords demonstrating the strongest citation bursts between 2005 and 2024.

CiteSpace was utilized to construct the keyword co-occurrence network (**Figure 8**). In the resulting graph, each node is indicative of a keyword. The size of a node corresponds to the frequency with which the associated keyword appears. Larger nodes signify higher frequencies of occurrence, whereas smaller nodes indicate lower frequencies. As shown in **Table 7**, the most common keywords include gastric cancer (567), artificial intelligence (165), surgery (163), deep learning (134), machine learning (132), lymph-node dissection (111), convolutional neural networks (100), classification (99), survival (97), laparoscopic gastrectomy (94), and outcomes (86). And the keywords with high centrality include cancer (0.22) and classification (0.15).

**Table 7:** Top 15 keywords in frequency, centrality and emergence intensity.

Rank	Keywords	Frequency	Keywords	Centrality	Keywords	Strength
1	gastric cancer	567	cancer	0.22	lymph node dissection	19.39
2	artificial intelligence	165	classification	0.15	assisted distal gastrectomy	12.7
3	surgery	163	distal gastrectomy	0.09	multicenter	11.85
4	deep learning	134	multicenter	0.09	subtotal gastrectomy	11.13
5	machine learning	132	resections	0.09	convolutional neural networks	10.75
6	lymph node dissection	111	lymphadenectomy	0.09	meta-analysis	7.81
7	convolutional neural networks	100	breast cancer	0.09	learning curve	7.44
8	classification	99	early gastric cancer	0.08	complications	7.35
9	survival	97	experience	0.08	robotic surgery	7.29
10	laparoscopic gastrectomy	94	expression	0.08	laparoscopic gastrectomy	6.97
11	outcome	86	ct	0.08	radical gastrectomy	6.74
12	robotic gastrectomy	82	gastric cancer	0.07	minimally invasive surgery	6.71
13	cancer	80	meta-analysis	0.07	resections	6.38
14	diagnosis	80	chemotherapy	0.07	surgery	6.15
15	assisted distal gastrectomy	71	neural networks	0.07	helicobacter pylori infection	4.93

Additionally, CiteSpace was applied to perform keyword clustering based on the Log-Likelihood Ratio algorithm (**Figure 8**). The modularity (Q = 0.5337 > 0.3) and silhouette score (S = 0.8204 > 0.7) indicate that the cluster structure is well-defined and the clustering results are reliable (26). A total of 11 clusters are identified in this study, and they are robotic gastrectomy, artificial intelligence, radiomics, deep learning, indocyanine green, breast cancer, decision tree, early gastric cancer, machine learning, and gastric adenocarcinoma.

**Figure 8** presents the emergence map depicting the 25 keywords exhibiting the most prominent bursts, among which the 15 with the greatest intensity are listed in **Table 7**. The keyword “lymph node dissection” exhibited a burst intensity of 19.39. The keyword “subtotal gastrectomy” (2007–2018) received the most sustained attention. In recent years, particularly between 2021 and 2024, the term upper gastrointestinal endoscopy has gained notable attention, while the phrase AI has become increasingly prevalent from 2022 to 2024. This trend indicates that subsequent investigations will probably place stronger emphasis on these emerging themes.

## 4 Discussion

### 4.1 Basic information

This investigation utilized bibliometric methods to examine research concerning the application of AI in GC between 2005 and 2024. The expansion of the literature in this area can be distinguished into two distinct stages. Before 2019, publication output grew gradually; aside from 2017 and 2018, which recorded 20 and 21 articles respectively, the annual number of papers remained below 20. From 2019 onward, however, publication activity increased dramatically, with more than 110 papers released each year from 2021 through 2024, peaking at 197 in 2024. These trends suggest that the role of AI in GC has attracted growing scholarly attention and has evolved into a major research priority. This surge may be attributed, in part, to the strategic emphasis placed on AI as a domain of international competition, supported by substantial investments and favorable policy initiatives worldwide. At the same time, the expanding integration of AI technologies into medical practice has highlighted their broad potential for advancing GC research (27). As a result, academic interest in applying AI to GC has intensified, thereby accelerating the development of this field.

With respect to publication output, China, Japan, South Korea, and the United States have produced the greatest volume of research, thereby establishing a leading position in this domain. The top three publications are all from Asian countries, which, on the one hand, is related to the high incidence of GC in these countries or regions, particularly in East Asia (28). On the other hand, it is closely related to the policies and financial support provided by these countries. For example, China has introduced a series of national AI plans to promote the development of this field (29). Notably, China and the United States were observed to have the highest TLS, while they ranked first and fourth, respectively, in publication output, thereby suggesting two distinct patterns of international cooperation and knowledge production. The American collaborative network, characterized by high TLS and relatively lower publication output, demonstrates a stronger orientation toward “quality” or “strength,” indicating that U.S. research teams tend to engage in deeper collaborations with leading international groups. The Chinese collaborative network, characterized by relatively higher TLS and publication output, reflects a strong “scale” orientation, suggesting that Chinese research teams exhibit high output efficiency and strong integrative capacity. These two modes of network cooperation play a significant role in advancing the development of this field.

Among the top 10 institutions in terms of publication volume, eight are from China, one is from Korea, and one is from Japan, which is relatively consistent with the distribution of publications across countries. Among these, Shanghai Jiao Tong University, Sun Yat-sen University, and the Chinese Academy of Sciences had the highest TLS, indicating that close cooperation was maintained. However, this cooperation is limited to domestic collaborations, and international cooperation and exchange remain restricted. Therefore, it is imperative to promote stronger international collaboration among institutions and to enhance their overall research competitiveness.

Academic journals are crucial for scholarly publications, and evaluating the number of publications in journals can assist researchers in selecting appropriate venues for manuscript submission. *Surgical Endoscopy and Other Interventional Techniques* published the highest number of papers, totaling 57. It is noteworthy that the top 10 academic journals, based on the number of publications, are primarily classified as Q1 and Q2, with 70% of journals in Q1 and 30% in Q2. The journal with the highest impact factor was *Gastrointestinal Endoscopy* (6.7), followed by *Gastric Cancer* (6.0). However, despite the significant contributions of Asian countries to research on AI applied to GC, only one Asian journal appears in the top 10, indicating underrepresentation. Therefore, the establishment of influential global journals within the Asian region is crucial to advancing the field.

### 4.2 Research hotspots and trends

Keywords serve as indicators of an article’s subject matter and central focus, and they frequently highlight prevailing research hotspots as well as emerging directions within a discipline (30). In the present analysis, the terms “gastric cancer” and “artificial intelligence” appeared with the highest frequency. It also included terms such as DL, ML, robotic gastrectomy, survival, outcomes, and diagnosis. This suggests that both the diagnosis and prognosis of AI-assisted GC are current research hotspots, which is highly consistent with the analysis of highly cited references. Furthermore, in this study, the keywords “upper gastrointestinal endoscopy” and “artificial intelligence” are identified as key terms expected to gain prominence by 2024, suggesting that AI-assisted upper gastrointestinal endoscopy diagnosis of GC is gradually gaining attention. Consequently, the primary areas of scholarly attention and the prevailing trends within this field can be summarized as follows.

#### 4.2.1 Machine learning and deep learning

According to cluster #3, deep learning, cluster #8, machine learning, and high-frequency keywords, both ML and DL are current research hotspots. As an important subset of AI, ML, which automatically develops mathematical algorithms from training data to make decisions without explicit programming, is widely utilized in the diagnosis and prognosis of gastric cancer (31, 32). A study based on ML algorithms, specifically random forest and LASSO regression, combined with bioinformatics analysis, identified four highly promising biomarkers that can be used to diagnose GC and predict overall survival (OS) in patients (33). Another study constructed a GC prognostic model based on four ML algorithms, including SVM-RFE, LASSO regression, ORSF, and XGBoost, which identified potential biomarkers to distinguish the molecular differences between cancer and normal tissues at the gene level, providing valuable insights into the pathogenesis and treatment of cancer (34). In addition, ML algorithms have been applied in the prediction of lymph node metastasis in GC. For example, studies on the development of prediction models based on ten ML algorithms have found that tumor invasion depth, smoking history, and lymphovascular invasion are independent risk factors for lymph node metastasis in gastric cancer, with the Gatboost model demonstrating strong predictive performance (35). All of these provide valuable references for clinical diagnosis and decision-making.

As an important branch of AI, DL has triggered revolutionary changes in medical image analysis. It overcomes the limitation of traditional ML, which requires manual definition of lesions, and can perform automatic feature learning while efficiently processing large amounts of data, offering advantages in stability and efficiency (36). The convolutional neural network is currently the most mature DL framework in the field of medical image analysis, particularly suited for image recognition and video analysis (37). It can automatically identify potential cancer based on the training with a large number of imaging images, significantly improving the early diagnosis of cancer, and demonstrating high accuracy in assessing the depth of gastric cancer invasion (38). Recent studies have shown that convolutional neural network-assisted systems exhibit high accuracy in the diagnosis of EGC and can enable novice endoscopists to perform at a diagnostic level comparable to that of expert endoscopists (39). In addition, DL models also exhibit strong accuracy in predicting treatment responses, such as responses to surgery, chemotherapy, and immunotherapy, and can identify populations that may benefit from these therapies, thus aiding in the development of personalized treatment regimens for GC patients (40, 41). It is evident that DL and ML are playing an increasingly important role in GC, having been applied in early diagnosis, treatment response, prognosis prediction, and other aspects, thereby providing valuable assistance to clinicians in better managing GC.

#### 4.2.2 Screening and diagnosis

Endoscopy is regarded as an important tool for the detection and screening of GC. Targeted biopsy, guided by endoscopic features, provides the basis for pathological diagnosis. However, the interpretation of gastroscopic images depends heavily on the clinical experience of endoscopists, and such interpretation may vary among individuals. Moreover, the workload associated with medical image analysis is substantial, making errors inevitable in routine practice. However, AI excels at processing and analyzing large datasets, and can assist clinical endoscopists in making diagnostic decisions and guiding biopsies, thereby enhancing both the accuracy and efficiency of diagnosis (3).

The results of one study indicated that the accuracy of an AI model in diagnosing GC reached 99.87%, substantially surpassing that of expert endoscopists (88.17%) (3). Gastro-MIL, an AI diagnostic model developed by Huang et al., was also reported to achieve high accuracy in GC diagnosis (42). An AI-based diagnostic system developed by Hirasawa et al. was capable of detecting cancers larger than 6 mm with an accuracy of 98.6% and a sensitivity of 92.2%, thereby demonstrating high diagnostic performance for GC lesions. This technique was recommended for clinical application to reduce the workload of endoscopists. However, this system exhibited a false-positive rate of 30.6% and failed to detect all cancer cases, particularly missing superficial depression and differentiated intramucosal cancers (25). In addition, Zhu et al. developed a convolutional neural network-based computer-aided diagnosis system and reported that it achieved high accuracy and specificity in diagnosing GC infiltration depth, significantly outperforming manual endoscopists (24). Several studies have further confirmed that AI diagnostic models demonstrate high accuracy, sensitivity, and specificity in GC diagnosis and hold broad application prospects in GC screening and diagnosis (43–45). With the efficient computational and learning capabilities of AI, diagnostic accuracy can be improved through the reduction of human error, while simultaneously decreasing physicians’ workload (46). AI-assisted diagnostic systems play a crucial role in treatment decision-making for GC (47). However, as clinical trials have not yet been conducted, their clinical feasibility and effectiveness remain to be established (48).

Globally, in countries or regions with limited medical resources and uneven distribution, AI diagnostic models can facilitate the feasibility of early screening and diagnosis of GC in low-resource settings, thereby bridging the diagnostic gap between countries and hospitals (8). In addition, although the early development and validation of AI models are costly, their large-scale application can reduce diagnostic costs and yield significant long-term benefits. In the future, with advances in 5G communication and AI technologies, the widespread adoption of EGC screening is anticipated, which may fundamentally reduce the incidence and mortality of GC.

#### 4.2.3 Prognosis prediction

Keyword analysis indicates that prognostic prediction has attracted considerable attention. Accurate prognostic prediction in clinical practice is of great importance for both physicians and patients. Predictive information enables physicians to make personalized clinical decisions that can improve patient survival rates and quality of life. However, prognostic outcomes are influenced by various factors, including pathological features, demographic characteristics, and physiological states of GC, and traditional statistical methods have difficulty analyzing the complex relationships among these variables (3). AI has demonstrated excellent performance in prognostic prediction of GC due to its strong learning and computational capabilities.

A retrospective study involving 2,320 patients applied a multi-task DL model based on preoperative CT images to predict peritoneal recurrence and disease-free survival, demonstrating that the model accurately predicted these outcomes in GC patients (32). An AI model employing a support vector machine (SVM) demonstrated superior predictive power for 5-year OS and disease-free survival after gastrectomy, achieving area under the curve (AUC) values of 0.773 and 0.751, respectively, compared with existing TNM staging systems (49). Another DL-based model, MIL-GC, also demonstrated strong performance in predicting OS among GC patients (42). In addition, InceptionV3, a DL-based AI model, performed effectively in predicting lymph node metastasis in EGC, achieving an accuracy of 79.44% and an AUC of 0.7181 (47). With continued advancements in AI technology, the accuracy of prediction models is expected to improve further, thereby enhancing their ability to assist clinicians in making personalized clinical decisions in the best interests of patients based on prognostic information.

However, several challenges remain in the application of AI in GC. First, interpretability and clinical trust are major concerns. AI models possess a “black box” feature (50), which limits their applicability in GC. The development and establishment of interpretable AI models enable researchers, clinicians, and patients to understand their operational processes, which strengthens trust in the diagnostic and predictive results of AI models for GC. Second, data quality and accountability are critical factors. The training and establishment of AI models depend on large datasets, where both data quality and noise can affect AI performance (51). Additionally, determining responsibility when AI fails or misdiagnoses is a key issue. The issue of accountability is particularly important (52). Secondly, promoting multi-center, large-scale clinical trials to verify the accuracy, stability, and feasibility of AI in GC diagnosis in real-world clinical settings, as well as its performance in prognosis prediction and its implementation in clinical practice, represents a core challenge and opportunity for future progress. Finally, AI also presents a double-edged sword. It is crucial to clearly define the role and positioning of AI in GC, which primarily serves to empower rather than replace physicians, with full consideration of the role of physicians in clinical applications (53). However, excessive reliance on AI may hinder physicians’ ability to think independently and make clinical judgments. Therefore, clinical physicians must strategically utilize AI, adhering to the principle of “physicians first, AI as auxiliary,” and should not overly rely on AI, to enhance physicians’ clinical capabilities and ultimately benefit GC patients.

### 4.3 Limitations

This study also has some shortcomings. First, only articles written in English from the Web of Science database were included in this study, which may have overlooked relevant literature from other databases. However, it is worth noting that the WoSCC database covers a wide range of subject areas and is one of the most widely used databases in bibliometrics, and visualization-based bibliometric analysis can provide researchers with a quick understanding of the field. Second, the latest published articles may not have been given enough attention and fully analyzed due to time factors, such as citation delays. Therefore, follow-up studies are needed to analyze them further.

## 5 Conclusion

In recent years, with the development of AI, the application of AI in GC has been increasing. This investigation provides a systematic and comprehensive examination of research outputs concerning the application of AI in GC over the past twenty years. The analysis revealed a consistent upward trajectory in annual publication volume, reflecting the increasing significance of this subject within the scholarly community. Among them, the most prolific institutions and countries are Yonsei University and China, respectively. *Surgical Endoscopy and Other Interventional Techniques* is the most active journal in the field, and Woo Jin Hyung is the most influential author. The analysis further emphasizes emerging focal areas and prevailing directions of research, such as the application of ML and DL techniques, along with the use of AI to support early detection, diagnostic evaluation, and prognostic assessment of GC. It can be expected that the application of AI in GC will become more widespread in the future. This trend is conducive to improving the diagnosis of EGC, assisting in the treatment and prognostic management of GC, and improving the survival rate and quality of life of patients.

## Data Availability

Data used or analyzed in this study are available from the corresponding author upon reasonable request.

## References

[1] Sung H Ferlay J Siegel RL Laversanne M Soerjomataram I Jemal A . Global cancer statistics 2020: GLOBOCAN estimates of incidence and mortality worldwide for 36 cancers in 185 countries. CA Cancer J Clin. (2021) 71:209–49. doi: 10.3322/caac.21660, PMID: 33538338

[2] Morgan E Arnold M Camargo MC Gini A Kunzmann AT Matsuda T . The current and future incidence and mortality of gastric cancer in 185 countries, 2020-40: A population-based modelling study. EClinicalMedicine. (2022) 47:101404. doi: 10.1016/j.eclinm.2022.101404, PMID: 35497064 PMC9046108

[3] Niu PH Zhao LL Wu HL Zhao DB Chen YT . Artificial intelligence in gastric cancer: Application and future perspectives. World J Gastroenterol. (2020) 26:5408–19. doi: 10.3748/wjg.v26.i36.5408, PMID: 33024393 PMC7520602

[4] Pattilachan TM Christodoulou M Ross S . Diagnosis to dissection: AI’s role in early detection and surgical intervention for gastric cancer. J Robot Surg. (2024) 18:259. doi: 10.1007/s11701-024-02005-6, PMID: 38900376

[5] Zhizhilashvili S Mchedlishvili I Camacho R Jankarashvili N Garuchava N Mebonia N . Descriptive epidemiology of gastric cancer: A population-based study from Georgia. Cureus. (2024) 16:e66862. doi: 10.7759/cureus.66862, PMID: 39280481 PMC11397424

[6] Alsallal M Habeeb MS Vaghela K Malathi H Vashisht A Sahu PK . Artificial intelligence in gastric cancer: a systematic review of machine learning and deep learning applications. Abdom Radiol (NY). (2025). doi: 10.1007/s00261-025-05181-7, PMID: 40932499

[7] Khosravi M Jasemi SK Hayati P Javar HA Izadi S Izadi Z . Transformative artificial intelligence in gastric cancer: Advancements in diagnostic techniques. Comput Biol Med. (2024) 183:109261. doi: 10.1016/j.compbiomed.2024.109261, PMID: 39488054

[8] Luo H Xu G Li C He L Luo L Wang Z . Real-time artificial intelligence for detection of upper gastrointestinal cancer by endoscopy: a multicentre, case-control, diagnostic study. Lancet Oncol. (2019) 20:1645–54. doi: 10.1016/S1470-2045(19)30637-0, PMID: 31591062

[9] Goto A Kubota N Nishikawa J Ogawa R Hamabe K Hashimoto S . Cooperation between artificial intelligence and endoscopists for diagnosing invasion depth of early gastric cancer. Gastric Cancer. (2023) 26:116–22. doi: 10.1007/s10120-022-01330-9, PMID: 36040575 PMC9813068

[10] Lei C Sun W Wang K Weng R Kan X Li R . Artificial intelligence-assisted diagnosis of early gastric cancer: present practice and future prospects. Ann Med. (2025) 57:2461679. doi: 10.1080/07853890.2025.2461679, PMID: 39928093 PMC11812113

[11] Reddy S Shaheed A Seo Y Patel R . Development of an artificial intelligence model for the classification of gastric carcinoma stages using pathology slides. Cureus. (2024) 16:e56740. doi: 10.7759/cureus.56740, PMID: 38650818 PMC11033212

[12] Zhang Z He T Huang L Li J Wang P . Immune gene prognostic signature for disease free survival of gastric cancer: Translational research of an artificial intelligence survival predictive system. Comput Struct Biotechnol J. (2021) 19:2329–46. doi: 10.1016/j.csbj.2021.04.025, PMID: 34025929 PMC8111455

[13] Pera M Gibert J Gimeno M Garsot E Eizaguirre E Miró M . Machine learning risk prediction model of 90-day mortality after gastrectomy for cancer. Ann Surg. (2022) 276:776–83. doi: 10.1097/SLA.0000000000005616, PMID: 35866643

[14] Lordick F Carneiro F Cascinu S Fleitas T Haustermans K Piessen G . Electronic address: clinicalguidelines@esmo.org. Gastric cancer: ESMO Clinical Practice Guideline for diagnosis, treatment and follow-up. Ann Oncol. (2022) 33(10):1005–20. doi: 10.1016/j.annonc.2022.07.004, PMID: 35914639

[15] Chen Y Wang B Zhao Y Shao X Wang M Ma F . Metabolomic machine learning predictor for diagnosis and prognosis of gastric cancer. Nat Commun. (2024) 15(1):1657. doi: 10.1038/s41467-024-46043-y, PMID: 38395893 PMC10891053

[16] Ke Y Tan C Zhen J Dong W . Global status and trends of gastric cancer and gastric microbiota research: a bibliometric analysis. Front Microbiol. (2024) 15:1341012. doi: 10.3389/fmicb.2024.1341012, PMID: 38655079 PMC11037409

[17] Jiang S Liu Y Zheng H Zhang L Zhao H Sang X . Evolutionary patterns and research frontiers in neoadjuvant immunotherapy: a bibliometric analysis. Int J Surg. (2023) 109:2774–83. doi: 10.1097/JS9.0000000000000492, PMID: 37216225 PMC10498839

[18] Yang Z Hotterbeex P Marent PJ Cerin E Thomis M van Uffelen J . Physical activity, sedentary behaviour, and cognitive function among older adults: A bibliometric analysis from 2004 to 2024. Ageing Res Rev. (2024) 97:102283. doi: 10.1016/j.arr.2024.102283, PMID: 38552882

[19] Song YP Liu JL Zong CZ Zhang FS Ren YF Ching YL . A bibliometric study on trends in chiropractic research from 1920 to 2023. Complement Ther Med. (2024) 82:103038. doi: 10.1016/j.ctim.2024.103038, PMID: 38582375

[20] Chen CM . CiteSpace II: Detecting and visualizing emerging trends and transient patterns in scientific literature. J Am Soc Inf Sci Technol. (2006) 57. doi: 10.1002/asi.20317

[21] van Eck NJ Waltman L . Software survey: VOSviewer, a computer program for bibliometric mapping. Scientometrics. (2010) 84:523–38. doi: 10.1007/s11192-009-0146-3, PMID: 20585380 PMC2883932

[22] Price DJS . Little science, big science. Columbia University Press (1965). 118 p. Available online at: https://book.douban.com/subject/26869791/.

[23] Bray F Ferlay J Soerjomataram I Siegel RL Torre LA Jemal A . Global cancer statistics 2018: GLOBOCAN estimates of incidence and mortality worldwide for 36 cancers in 185 countries. CA Cancer J Clin. (2018) 68:394–424. doi: 10.3322/caac.21492, PMID: 30207593

[24] Zhu Y Wang QC Xu MD Zhang Z Cheng J Zhong YS . Application of convolutional neural network in the diagnosis of the invasion depth of gastric cancer based on conventional endoscopy. Gastrointest Endosc. (2019) 89:806–815.e1. doi: 10.1016/j.gie.2018.11.011, PMID: 30452913

[25] Hirasawa T Aoyama K Tanimoto T Ishihara S Shichijo S Ozawa T . Application of artificial intelligence using a convolutional neural network for detecting gastric cancer in endoscopic images. Gastric Cancer. (2018) 21:653–60. doi: 10.1007/s10120-018-0793-2, PMID: 29335825

[26] Liu W Liu W Huang L Guo Z Liang Y Zhang H . Hotspots and trends in acupuncture-assisted tumor chemotherapy:a bibliometric analysis based on CiteSpace and VOSviewer. Chin Acupuncture Moxibustion. (2024) 44:1453–62. doi: 10.13703/j.0255-2930.20231229-k0005, PMID: 39658387

[27] Hirasawa T Ikenoyama Y Ishioka M Namikawa K Horiuchi Y Nakashima H . Current status and future perspective of artificial intelligence applications in endoscopic diagnosis and management of gastric cancer. Dig Endosc. (2021) 33:263–72. doi: 10.1111/den.13890, PMID: 33159692

[28] He Y Wang Y Luan F Yu Z Feng H Chen B . Chinese and global burdens of gastric cancer from 1990 to 2019. Cancer Med. (2021) 10:3461–73. doi: 10.1002/cam4.3892, PMID: 33931958 PMC8124120

[29] Wang FS Liu L . Exploration and practice of general artificial intelligence basic course teaching. Comput Educ. (2021), 15–19. doi: 10.16512/j.cnki.jsjjy.2021.10.005

[30] Gao M Zhang H Gao Z Sun Y Wang J Wei F . Global hotspots and prospects of perimenopausal depression: A bibliometric analysis via CiteSpace. Front Psychiatry. (2022) 13:968629. doi: 10.3389/fpsyt.2022.968629, PMID: 36164290 PMC9508326

[31] Arai J Aoki T Sato M Niikura R Suzuki N Ishibashi R . Machine learning-based personalized prediction of gastric cancer incidence using the endoscopic and histologic findings at the initial endoscopy. Gastrointest Endosc. (2022) 95:864–72. doi: 10.1016/j.gie.2021.12.033, PMID: 34998795

[32] Jiang Y Zhang Z Yuan Q Wang W Wang H Li T . Predicting peritoneal recurrence and disease-free survival from CT images in gastric cancer with multitask deep learning: a retrospective study. Lancet Digital Health. (2022) 4:e340–e350. doi: 10.1016/S2589-7500(22)00040-1, PMID: 35461691

[33] Liu Y Bian B Chen S Zhou B Zhang P Shen L . Identification and validation of four serum biomarkers with optimal diagnostic and prognostic potential for gastric cancer based on machine learning algorithms. Cancer Med. (2025) 14:e70659. doi: 10.1002/cam4.70659, PMID: 40084401 PMC11907202

[34] Zhang ZJ Han KS Yang M . Identification of critical genes and construction of a prognostic model for stomach adenocarcinoma using machine learning algorithms. Genomics Appl Biol. (2025) 44:826–35. doi: 10.13417/j.gab.044.000826

[35] Meng XY Qin JY Chen WS . Construction and validation of a prediction model for lymph node metastasis in early gastric cancer based on machine learning. J Army Med Univ. (2024) 46:2432–42. doi: 10.16016/j.2097-0927.202403126

[36] Zhang Q Cao YT Wang ZJ Zhou BQ . Advances in deep learning for endoscopic image-based diagnosis of early gastric cancer. J Pract Med. (2025) 41:2160–6. doi: 10.3969/j.issn.1006-5725.2025.14.006

[37] Okagawa Y Abe S Yamada M Oda I Saito Y . Artificial intelligence in endoscopy. Dig Dis Sci. (2022) 67:1553–72. doi: 10.1007/s10620-021-07086-z, PMID: 34155567

[38] Xie F Zhang K Li F Ma G Ni Y Zhang W . Diagnostic accuracy of convolutional neural network-based endoscopic image analysis in diagnosing gastric cancer and predicting its invasion depth: a systematic review and meta-analysis. Gastrointestinal endoscopy. (2022) 95(4):599–609. doi: 10.1016/j.gie.2021.12.021, PMID: 34979114

[39] Feng J Zhang Y Feng Z Ma H Gou Y Wang P . A prospective and comparative study on improving the diagnostic accuracy of early gastric cancer based on deep convolutional neural network real-time diagnosis system (with video). Surg Endosc. (2025) 39:1874–84. doi: 10.1007/s00464-025-11527-5, PMID: 39843600

[40] Zhang J Zhang Q Zhao B Shi G . Deep learning nomogram for predicting neoadjuvant chemotherapy response in locally advanced gastric cancer patients. Abdom Radiol (NY). (2024) 49:3780–96. doi: 10.1007/s00261-024-04331-7, PMID: 38796795 PMC11519172

[41] Sang S Sun Z Zheng W Wang W Islam MT Chen Y . TME-guided deep learning predicts chemotherapy and immunotherapy response in gastric cancer with attention-enhanced residual Swin Transformer. Cell Rep Med. (2025) 6:102242. doi: 10.1016/j.xcrm.2025.102242, PMID: 40695288 PMC12432364

[42] Huang B Tian S Zhan N Ma J Huang Z Zhang C . Accurate diagnosis and prognosis prediction of gastric cancer using deep learning on digital pathological images: A retrospective multicentre study. EBioMedicine. (2021) 73:103631. doi: 10.1016/j.ebiom.2021.103631, PMID: 34678610 PMC8529077

[43] Li L Chen Y Shen Z Zhang X Sang J Ding Y . Convolutional neural network for the diagnosis of early gastric cancer based on magnifying narrow band imaging. Gastric Cancer. (2020) 23:126–32. doi: 10.1007/s10120-019-00992-2, PMID: 31332619 PMC6942561

[44] Noda H Kaise M Higuchi K Koizumi E Yoshikata K Habu T . Convolutional neural network-based system for endocytoscopic diagnosis of early gastric cancer. BMC Gastroenterol. (2022) 22:237. doi: 10.1186/s12876-022-02312-y, PMID: 35549679 PMC9102244

[45] Ueyama H Kato Y Akazawa Y Yatagai N Komori H Takeda T . Application of artificial intelligence using a convolutional neural network for diagnosis of early gastric cancer based on magnifying endoscopy with narrow-band imaging. J Gastroenterol Hepatol. (2021) 36:482–9. doi: 10.1111/jgh.15190, PMID: 32681536 PMC7984440

[46] Yu KH Beam AL Kohane IS . Artificial intelligence in healthcare. Nat BioMed Eng. (2018) 2:719–31. doi: 10.1038/s41551-018-0305-z, PMID: 31015651

[47] Yang R Zhang J Zhan F Yan C Lu S Zhu Z . Artificial intelligence efficiently predicts gastric lesions, Helicobacter pylori infection and lymph node metastasis upon endoscopic images. Chin J Cancer Res. (2024) 36:489–502. doi: 10.21147/j.issn.1000-9604.2024.05.03, PMID: 39539812 PMC11555197

[48] Hsiao YJ Wen YC Lai WY Lin YY Yang YP Chien Y . Application of artificial intelligence-driven endoscopic screening and diagnosis of gastric cancer. World J Gastroenterol. (2021) 27:2979–93. doi: 10.3748/wjg.v27.i22.2979, PMID: 34168402 PMC8192292

[49] Li X Zhai Z Ding W Chen L Zhao Y Xiong W . An artificial intelligence model to predict survival and chemotherapy benefits for gastric cancer patients after gastrectomy development and validation in international multicenter cohorts. Int J Surg. (2022) 105:106889. doi: 10.1016/j.ijsu.2022.106889, PMID: 36084807

[50] Wang F Kaushal R Khullar D . Should health care demand interpretable artificial intelligence or accept “Black box” Medicine? Ann Intern Med. (2020) 172:59–60. doi: 10.7326/M19-2548, PMID: 31842204

[51] Kleinberg J Ludwig J Mullainathan S Sunstein CR . Discrimination in the age of algorithms. J Legal Anal. (2018) 10:113–74. doi: 10.1093/jla/laz001

[52] Aung YYM Wong DCS Ting DSW . The promise of artificial intelligence: a review of the opportunities and challenges of artificial intelligence in healthcare. Br Med Bull. (2021) 139:4–15. doi: 10.1093/bmb/ldab016, PMID: 34405854

[53] Topol EJ . High-performance medicine: the convergence of human and artificial intelligence. Nat Med. (2019) 25:44–56. doi: 10.1038/s41591-018-0300-7, PMID: 30617339

